# Supplementary material for: Phenotypic identification of Metallo-ß- lactamase resistance Gram negative bacteria from a clinical specimen in Sidama, Ethiopia
Source: PLoS One. 2025 Jan 8;20(1):e0313431. doi: 10.1371/journal.pone.0313431 (PMC11709301; doi:10.1371/journal.pone.0313431)
Supplement: Attachment — Submitted filename: Decision PONE-D-24-02706.docx [file pone.0313431.s002.docx]

The manuscript titled “Phenotypic Identification of Metallo-ß- lactamase resistance Gram Negative bacteria from a clinical specimen in Sidama, Ethiopia” is important in that the authors have highlighted the detection of Carbapenemase and MBL producers by phenotypes of Gram-negative bacteria in Sidama, Ethiopia. Undoubtedly the study has a profound impact as the WHO (2017) highlighted drug-resistant GNB as a priority pathogen.

It can be understood that there is no laboratory molecular setup in developing countries to perform confirmatory tests. As well as low-budget research it is difficult to do molecular tests. However, the study has several deficits, and gaps without addressing these concerns the manuscript is not recommended for publication.
The authors have shown only Carbapenem resistance without testing tigecycline and polymyxin-b how EDR and PDR strain detection which is unclear in the methods section.

It is important to learn whether these stains have colistin or other last-resort antibacterial agents’ resistance.

There are several recent papers the authors have missed, there is no comparison or discussion between these papers and the author’s findings.
The discussion should be improved and the conclusion should be revised.

It makes no sense merely comparing percentage occurrence of one trait or phenotype with other studies. The methods used to detect these traits may be different for the previous studies. Just comparing if the percent occurrence in the present study was similar to, or greater or smaller than the previous study does not suggest anything in the current frame of discussion. The authors need to elaborate more on the results obtained in the present study and draw inferences from the same.

Research results should be highlighted as the major outcome and recommendation is required.
