# Supplementary material for: Phenotypic identification of Metallo-ß- lactamase resistance Gram negative bacteria from a clinical specimen in Sidama, Ethiopia
Source: PLoS One. 2025 Jan 8;20(1):e0313431. doi: 10.1371/journal.pone.0313431 (PMC11709301; doi:10.1371/journal.pone.0313431)
Supplement: Attachment — Submitted filename: Point by Point Response.docx [file pone.0313431.s003.docx]

**Point by point response**

**General Response**

**Dear Editor and reviewers, thank you very much for your expertise and time to review and edit our manuscript. As per your recommendation we modify and include the data set as supporting file. Here we tried to make a modification based on your comments. Even if, the reviewer 1 comments are not specific and difficult to address as per your comments we tried to address a general response. For reviewer 2 since his comment is specific, we tried to address point by point and shaded on the manuscript with yellow color for both modifications.**

**Reviewer 1:**

**Comment**: The manuscript titled “Phenotypic Identification of Metallo-ß- lactamase resistance Gram Negative bacteria from a clinical specimen in Sidama, Ethiopia” is important in that the authors have highlighted the detection of Carbapenemase and MBL producers by phenotypes of Gram-negative bacteria in Sidama, Ethiopia. Undoubtedly the study has a profound impact as the WHO (2017) highlighted drug-resistant GNB as a priority pathogen.

**Response: Thank for recognizing our work**

**Comment**: It can be understood that there is no laboratory molecular setup in developing countries to perform confirmatory tests. As well as low-budget research it is difficult to do molecular tests. However, the study has several deficits, and gaps without addressing these concerns the manuscript is not recommended for publication.
The authors have shown only Carbapenem resistance without testing tigecycline and polymyxin-b how EDR and PDR strain detection which is unclear in the methods section. It is important to learn whether these stains have colistin or other last-resort antibacterial agents’ resistance.

**Response: We write this idea in the limitation but the CLSI recommend as to check the presence of Carbapenem resistance phenotypically with carbapenem class of antibiotics. At this time, we couldn’t improve as per your comment. I hope you understand the shortage of antibiotics in the local market.**

**Comment:** There are several recent papers the authors have missed, there is no comparison or discussion between these papers and the author’s findings.
The discussion should be improved and the conclusion should be revised.

It makes no sense merely comparing percentage occurrence of one trait or phenotype with other studies. The methods used to detect these traits may be different for the previous studies. Just comparing if the percent occurrence in the present study was similar to, or greater or smaller than the previous study does not suggest anything in the current frame of discussion. The authors need to elaborate more on the results obtained in the present study and draw inferences from the same.

**Response:** We tried to modify the discussion as per your comment and add a justification.

**Comment:** Research results should be highlighted as the major outcome and recommendation is required.

**Response: We tried to modify the conclusion and recommendation.**

**Reviewer #2:**

**Comment:**

**In Page 1:**

City and country of each author should be added to the Authors’ affiliation.

**Response: modified as per your comment**

**Comment:**

Abstract:
• Page 1: Method section needs more explanation about the practical work.

**Response: modified**

• Page 2, lines 40-42: The sentence “The rates of multi, extensive and pan-drug resistance bacteria accounted for 83.7%, 50.3%, and 17.0%, respectively” needs more explanation. What do these numbers mean?

**Response**: modified

**Comments:**

Introduction
• Page 3, Line 67: What does the abbreviation “ESBLs” mean?

**Response:** modified

• Page 3, Line 89: Correct “20.9 o C”.

**Response**: modified

**Comments:**

Materials and Methods

• Page 4, line 101: delete “&”.

**Response: modified**

• Page 4, line 110: delete “Study Methodology”

**Response**: Accepted

• Page 4, line 125: correct “37 C0”

**Response: modified**

• Page 5, line 142: correct the sentence “Twelve different antibacterial will be performed” to be “Twelve different antibacterial agents were used”

**Response: modified**

.• Page 5, line 142: Write the full name of “mCIM” and similar abbreviations in other pages.

**Response: modified**

• Page 6, lines 186, 187: the sentence “Gram-negative bacilli that produced serine carbapenemase were those that were carbapenemase positive but Metallo carbapenemase negative” is not necessary.

**Response: modified**

• Page 6, line 195: add the IRB number.

**Response: modified**

**Comments**

Results:
• Some typing errors need correction in the whole results section, specifically spaces between numbers.

**Response: modified**

• Page 7, line 225: what do you mean by this sentence “from this most of them 226 used more than two types of external devices 62 (40.5%)”?

**Response: this is modified**

• Page 8: abbreviations of the antibiotics used should be added to the Table 1 capture.

**Response: modified**

• Page 10, line 259: correct the word “statical” to be “statistical”.

**Response: modified**

**Comments**

Discussion:
• Please remove some “comma symbol where is not necessary.

**Response: modified**

• Please revise the text without “Italic” typing other than the name of bacteria.

**Response: modified**

• Write the full name for the abbreviations.

**Response: modified**

•Some typing errors should be revised.

**Response: modified**

**Comments**

Conclusion:

• Page 13, line 323: correct the word “Impenem” to “imipenem”

**Response: modified**

**The end**
